# A Rare Presentation of Ascites Secondary to Inferior Vena Cava Thrombosis in a Patient With Factor V Leiden Mutation

**DOI:** 10.7759/cureus.61973

**Published:** 2024-06-08

**Authors:** Sunpil Hwang, Dania Kaur, Mina Iskander, Philip Botelho, Valvani Rachna

**Affiliations:** 1 Internal Medicine, North Alabama Medical Center, Florence, USA

**Keywords:** venous thrombosis, venous angioplasty, unexplained ascites, inferior vena cava thrombus, factor v leiden (fvl)

## Abstract

We present the case of a 36-year-old female with Factor V Leiden mutation taking warfarin, who presented to the emergency department with swelling in the abdominal and bilateral lower extremities. Initial assessment revealed an international normalized ratio (INR) of 5.0. Abdomen/pelvis computed tomography (CT) and computed tomographic angiography (CTA) scans indicated chronic thrombosis of the inferior vena cava (IVC), leading to the development of ascites and swelling. Extensive investigations were conducted to explore potential contributing factors for the ascites and edema, all of which yielded negative results. Warfarin was discontinued, and unfractionated heparin was initiated once the INR decreased to 2.0. The patient underwent IVC angioplasty with stent placement, resulting in significant improvement of ascites and lower extremity swelling. Subsequently, heparin was transitioned to oral warfarin, and therapeutic INR levels were achieved before discharge. At the follow-up outpatient visit, the patient's ascites and lower extremity edema had completely resolved. This case highlights a rare instance of IVC involvement associated with Factor V Leiden mutation. Furthermore, the patient's history of noncompliance with medication, initial supratherapeutic INR, and chronic IVC thrombosis emphasize the importance of medication adherence and the crucial role of primary care in ensuring regular follow-up and monitoring.

## Introduction

Factor V Leiden mutation represents one of the most prevalent inherited thrombophilic disorders among individuals of European descent [1,2]. This condition follows an autosomal dominant inheritance pattern. This genetic anomaly confers resistance upon affected individuals to activated protein C (APC) due to a mutation in the Factor Va gene, thereby increasing their susceptibility to thromboembolic events [3]. Typically, patients with Factor V Leiden mutation manifest with venous thromboembolism (VTE), most commonly affecting the deep vein system of the lower extremities and pulmonary vasculature. However, in exceptional instances, thrombosis may occur in cerebral dural sinuses, retinal veins, hepatic veins, or portal veins. Involvement of the inferior vena cava (IVC) secondary to Factor V Leiden mutation is exceedingly rare. Here, we present a compelling case report illustrating chronic IVC thrombosis precipitated by Factor V Leiden mutation, resulting in the formation of ascites and anasarca in the absence of underlying hepatic or cardiac pathology.

## Case presentation

A 36-year-old female with a past medical history of Factor V Leiden with an unspecified genotype on warfarin therapy presented to the emergency department due to progressive abdominal and bilateral lower extremity swelling. The patient reported that the swelling had initially started in her legs a few months ago and subsequently progressed to involve her abdomen over the past few weeks. She also reported experiencing shortness of breath, nausea, abdominal pain, and decreased appetite. She denied any episodes of fever, chills, or chest pain. She also mentioned that she had not been taking her medication as prescribed. Her vital signs on presentation were within normal limits, except for a sinus tachycardia with a heart rate of 118 beats per minute. Upon physical examination, her abdomen was distended but non-tender. There were no clinical signs of cirrhosis, such as icteric sclera or spider angioma. Initial laboratory results are presented below in Table 1.

**Table 1:** Initial laboratory results on presentation INR: international normalized ratio

Lab parameter	Values	Reference range
Hemoglobin	12.2 g/dL	12.0-16.0 g/dL
White blood cell	4.8×10^9^/L	4.3-11.0 ×10^9^/L
Platelet	181×10^9^/L	150-375×10^9^/L
Prothrombin time	10.9 seconds	9.0-11.6 seconds
INR	5.0	0.8-1.2
Sodium	135 mmol/L	135-145 mmol/L
Potassium	3.0 mmol/L	3.6-5.2 mmol/L
Carbon dioxide	29 mmol/L	21-32 mmol/L
Anion gap	8	6-18
Creatinine	0.4 mg/dL	0.6-1.3 mg/dL
B-Natriuretic peptide	223 pg/mL	<300 pg/mL
Albumin	2.8 g/dL	3.4-5.0 g/dL
Alanine transaminase	18 U/L	0-35 U/L
Aspartate transaminase	63 U/L	0-59 U/L
Alkaline phosphatase	190 U/L	32-104U/L
Total bilirubin	0.80 mg/dL	0-1.0 mg/dL
Lipase	<10 U/L	23-300 U/L
Hepatitis A IgM antibody	Nonreactive	Nonreactive
Hepatitis B surface antigen	Nonreactive	Nonreactive
Hepatitis B surface antibody	Nonreactive	Nonreactive
Hepatitis C surface antibody	Nonreactive	Nonreactive
HIV 1 and 2 antibody	Nonreactive	Nonreactive

**Figure 1:**
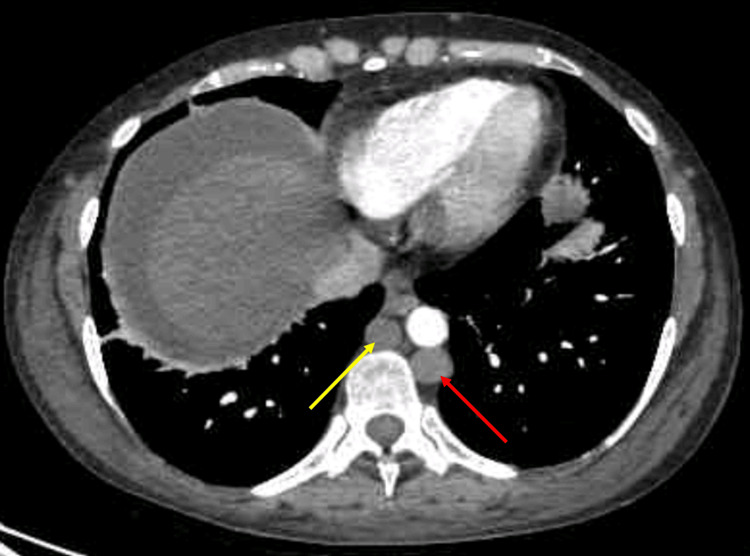
Abdominal/pelvis CTA showing the azygous-hemiazygous system Depicts the azygous-hemiazygous system, with a red arrow denoting the hemiazygous vein and a yellow arrow indicating the azygous vein. These prominent venous systems emerged as a consequence of IVC occlusion secondary to thrombosis. IVC: inferior vena cava; CTA: computed tomographic angiography

**Figure 2:**
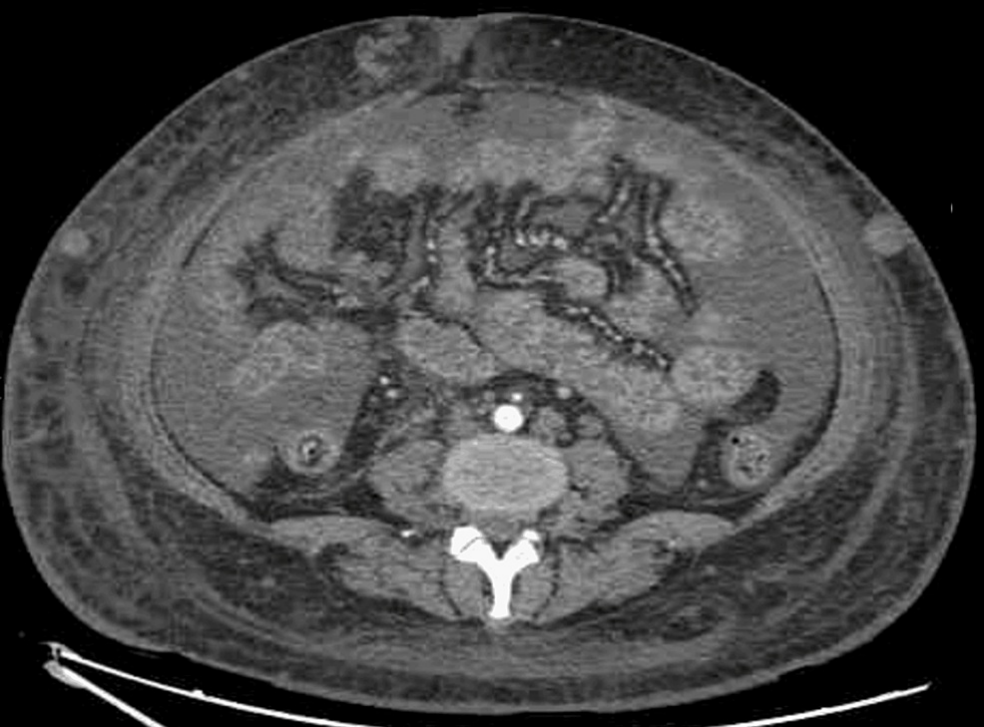
Abdominal/pelvis CTA showing ascites and subcutaneous edema Illustrates extensive edema within the abdominal wall along with subcutaneous collateral vessels as well as the presence of ascites. CTA: computed tomographic angiography

**Figure 3:**
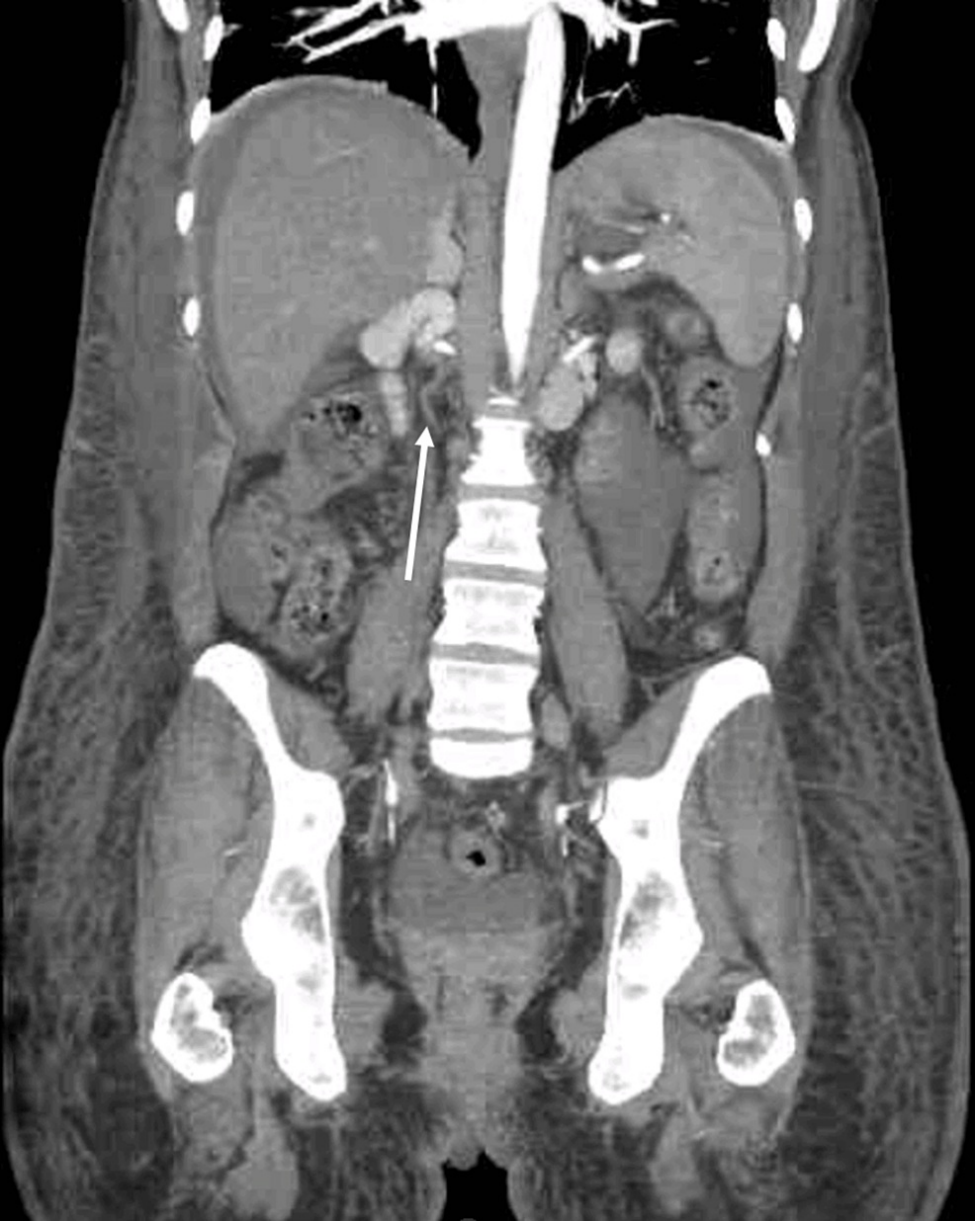
Coronal abdominal/pelvis CTA Illustrates a hypoplastic IVC below the confluence of the renal vein, as denoted by a white arrow. CTA: computed tomographic angiography; IVC: inferior vena cava

**Figure 4:**
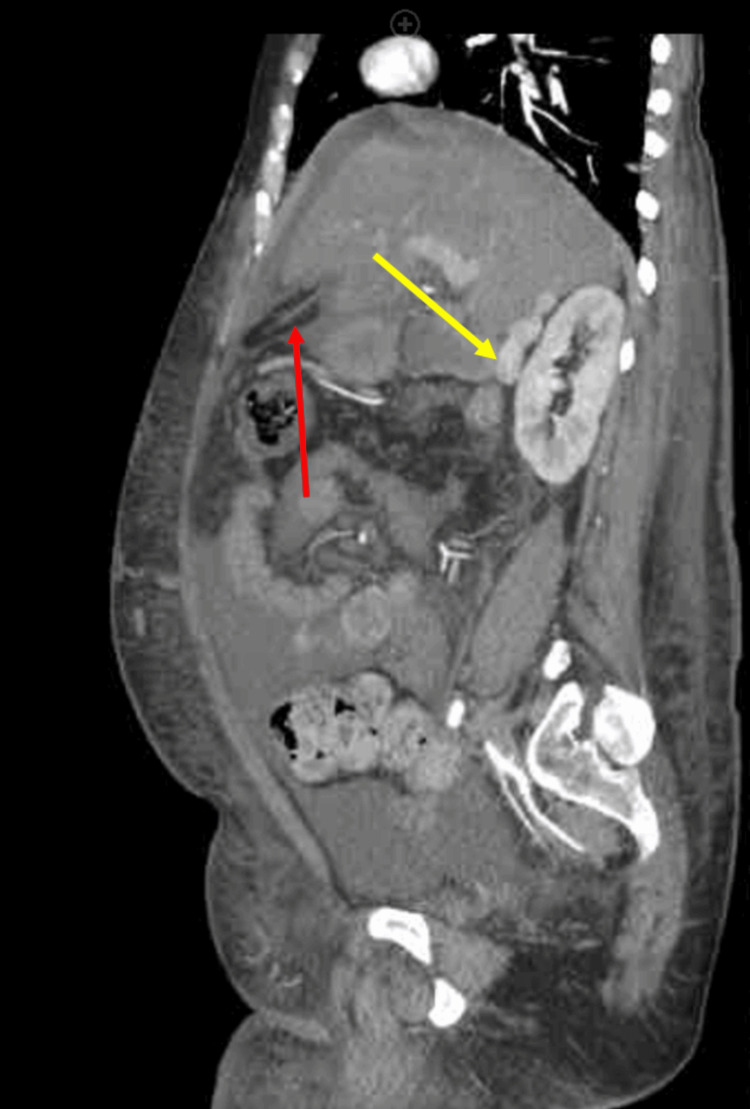
Sagittal abdominal/pelvis CTA Demonstrates recanalization of the umbilical vein from IVC occlusion, highlighted by a red arrow. A yellow arrow denotes a supra-renal IVC. A hypoplastic infrarenal IVC is observed below this point. CTA: computed tomographic angiography; IVC: inferior vena cava

The chest X-ray was negative for an acute cardiopulmonary process. Doppler ultrasound of the lower extremity was negative for deep vein thrombosis (DVT). However, an abdominal/pelvic computed tomography (CT) scan revealed a hypoplastic IVC with multiple collateral vessels in the azygos-hemiazygos system as well as on the abdominal wall. A moderate amount of ascites was also noted. Subsequent abdominal/pelvic computed tomographic angiography (CTA) showed an atrophic appearance of the inferior portion of the IVC with multiple small collateral vessels, consistent with chronic occlusion of the IVC, likely from thrombosis (Figures 1-4). The narrowing was observed in the renal and hepatic portions of the IVC, which remained patent. No signs of liver cirrhosis were noted on either imaging. Echocardiography revealed an ejection fraction of 55%, along with unremarkable diastolic function and the absence of significant valvular disease.

The patient was managed medically with oral furosemide 40 mg once daily and oral spironolactone 100 mg once daily for moderate ascites. The vascular surgery team was consulted regarding the occlusion of the IVC and planned to perform an angioplasty. Warfarin therapy was discontinued to reduce the supratherapeutic international normalized ratio (INR) to a safe level for the procedure. Once the INR decreased to 2.0, unfractionated heparin was initiated until the procedure date. Meanwhile, comprehensive laboratory tests were conducted to exclude any secondary etiologies of ascites and anasarca. Autoimmune antibody tests, including anti-nuclear antibody (ANA), cytoplasmic anti-neutrophil cytoplasmic antibody (c-ANCA), perinuclear anti-neutrophil cytoplasmic antibody (p-ANCA), anti-mitochondrial antibody (AMA), and smooth muscle antibody (SMA), were all negative. Alpha-fetoprotein level was within the normal range at 2.330 ng/mL (reference range: <7.51 ng/mL), and alpha-1 antitrypsin level also fell within the normal range at 170 mg/dL (reference range: 100-188 mg/dL). Only ceruloplasmin was noted to be slightly low at 17.7 mg/dL (reference range: 19.0-39.0 mg/dL). However, the patient did not exhibit any other neurological or psychiatric symptoms, nor did she present with Kayser-Fleischer rings. Additionally, she denied any family history of Wilson’s disease.

**Figure 5:**
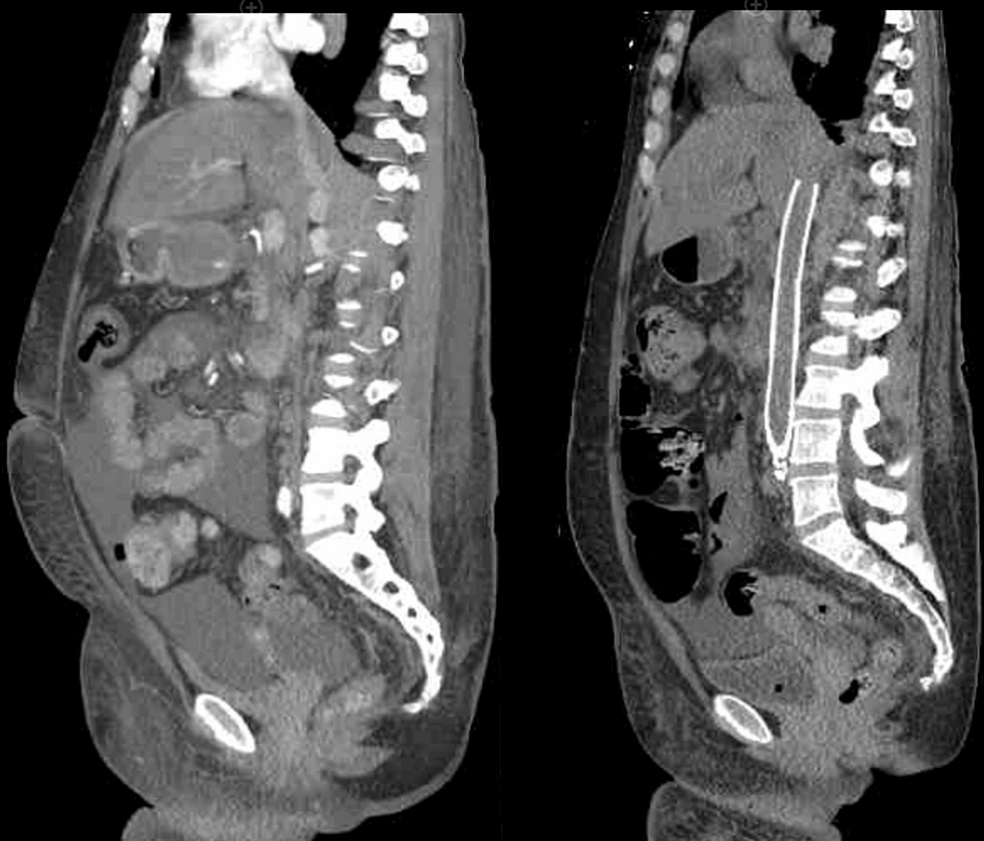
Sagittal abdominal/pelvis CT pre- and post-angioplasty Presents a sagittal image of the abdomen/pelvis, comparing pre-angioplasty (left, CTA) and post-angioplasty (right, CT without contrast). Following angioplasty, the CT image on the right demonstrates a notable reduction in anasarca and the volume of ascites. CTA: computed tomographic angiography; CT: computed tomography

The patient subsequently underwent angioplasty of the IVC with stent placement, which was performed without complications. Following the procedure, warfarin was reinitiated with heparin bridging, targeting an INR of 3.0. Additionally, aspirin 81 mg and clopidogrel 75 mg were added. The patient exhibited significant improvement in abdominal and lower extremity edema. A repeat abdominal/pelvic CT scan without contrast demonstrated marked improvement in ascites and anasarca (Figure 5). The patient was discharged with instructions for further follow-up with the vascular surgery team to optimize warfarin therapy and monitor INR levels. At the recent follow-up visit, the patient's edema was noted to be completely resolved.

## Discussion

Factor V Leiden is the most common inherited form of thrombophilia. Its prevalence varies by ethnicity, rare in Asian, African, and indigenous Australian populations. In contrast, it is more prevalent in the white population, with reported prevalence ranging from 1% to 5% [1,2]. Factor V Leiden is characterized by a reduced anticoagulant function of APC. APC is a natural anticoagulant that works by inactivating factors Va and VIIIa, thereby slowing down the coagulation process in the body [4]. In individuals with Factor V Leiden, APC is unable to effectively inactivate Factor Va due to a single point mutation (guanine to adenine at nucleotide 1691), resulting in the substitution of arginine with glutamine at position 506 (Arg506Gln) in the cleavage site of Factor Va where APC acts. This mutation leads to Factor Va being resistant to APC, causing a 10-fold prolongation in the inactivation rate of Factor Va [1,5,6]. This altered mechanism increases the risk of thrombosis.

While the primary clinical manifestation of Factor V Leiden is VTE, many individuals with this mutation do not develop VTE as anticipated. In the case of heterozygous Factor V Leiden, only about 10% of individuals will experience VTE during their lifetime [7]. A study indicated that individuals with homozygous Factor V Leiden typically experience their first episode of VTE by the age of 33 years. In contrast, heterozygotes and unaffected individuals have a 20% and 8% risk, respectively [8]. Among patients with Factor V Leiden who do develop VTE, the most common form is DVT of the lower extremity. Pulmonary embolism is also a frequently observed type of VTE in this population. Additionally, thrombosis at unusual sites has been reported, including involvement of the superficial veins of the legs, cerebral veins, portal veins, and retinal veins [9-12].

IVC thrombosis is a highly uncommon condition, it has been associated with various factors, including congenital anomalies of the IVC, compression by adjacent masses, pregnancy, or post-endovascular IVC procedures. Among thrombophilic disorders, antiphospholipid syndrome has been observed to be more frequently associated with IVC thrombosis than any other disorder [13]. The prevalence of IVC thrombosis specifically attributable to Factor V Leiden is not well-defined due to the extreme rarity of the condition.

The patient, in this case, presented with abdominal and bilateral lower extremity swelling and had a known medical history of Factor V Leiden with an unspecified genotype. Further evaluation with abdominal/pelvic CT and CTA scans revealed ascites without evidence of cirrhosis but with signs suggestive of chronic IVC thrombosis. Despite the presumed association between the patient's anasarca and IVC occlusion, an extensive diagnostic workup was conducted to exclude secondary causes.

The patient's liver function tests were unremarkable, and hepatitis panel tests were negative. The patient exhibited a mildly decreased level of albumin at 2.8 g/dL. Nevertheless, she lacked any established risk factors associated with cirrhosis, such as a history of alcohol consumption or obesity. Furthermore, the liver function test results and CT imaging failed to manifest any discernible signs of cirrhosis. Given the substantial volume of ascites present, any signs of underlying cirrhosis would reasonably be expected to be observed through both imaging and laboratory analyses [14].

Potential autoimmune diseases, including autoimmune hepatitis and primary biliary cirrhosis, were ruled out based on negative results for ANA, c-ANCA, p-ANCA, AMA, and SMA. The alpha-fetoprotein and alpha-1 antitrypsin levels were also within the normal range. Although the level of ceruloplasmin was slightly reduced, the absence of other signs of Wilson's disease, such as cirrhosis, neurological symptoms (e.g., gait abnormalities or parkinsonian symptoms), psychiatric symptoms (e.g., depression or psychosis), and Kayser-Fleischer rings, made the likelihood of Wilson's disease in this patient very low. Furthermore, the resolution of the patient's symptoms following angioplasty strongly supports the conclusion that the ascites and lower extremity swelling were attributable to chronic IVC thrombosis secondary to Factor V Leiden.

Additionally, it is noteworthy that the patient presented with a markedly elevated INR of 5.0. This observation suggests the patient's non-compliance with warfarin therapy and irregular follow-up with her primary care provider. Given the presence of chronic IVC thrombosis on CT imaging, a condition that should have been mitigated with proper warfarin adherence, it is more plausible to ascertain the patient's non-adherence to treatment. This rationale is further corroborated by the patient's self-reported failure to adhere to medication instructions.

This case highlights the importance of medication adherence and the role of primary care in managing patients taking medications necessitating regular monitoring and follow-ups. Medication non-adherence poses a significant challenge in clinical practice, particularly with medications like warfarin that require close monitoring [15]. Patients should be educated about the importance of adherence to prescribed medications and encouraged to regularly follow-up with their primary care physicians for monitoring and adjustments, which may help prevent adverse events resulting from non-adherence, as illustrated in this case.

## Conclusions

This case report presents an intriguing and rare scenario in which Factor V Leiden mutation results in chronic IVC thrombosis, manifesting as ascites and bilateral lower extremity swelling. We anticipate that this case report will increase awareness among medical societies regarding its potential to cause such severe complications. Furthermore, this case emphasizes the critical importance of medication adherence and the role of primary care, particularly for medications requiring close monitoring, such as warfarin, as demonstrated here.

## References

[1] Kujovich JL (2011). Factor V Leiden thrombophilia. Genet Med.

[2] Dzimiri N, Meyer B (1996). World distribution of factor V Leiden. Lancet.

[3] Yusuf M, Gupta A, Kumar A, Afreen S (2012). Mechanism and pathophysiology of activated protein C-related factor V leiden in venous thrombosis. Asian J Transfus Sci.

[4] Dahlbäck B (2008). Advances in understanding pathogenic mechanisms of thrombophilic disorders. Blood.

[5] Simioni P, Prandoni P, Lensing AW (1997). The risk of recurrent venous thromboembolism in patients with an Arg506-->Gln mutation in the gene for factor V (factor V Leiden). N Engl J Med.

[6] Thorelli E, Kaufman RJ, Dahlbäck B (1999). Cleavage of factor V at Arg 506 by activated protein C and the expression of anticoagulant activity of factor V. Blood.

[7] Heit JA, Sobell JL, Li H, Sommer SS (2005). The incidence of venous thromboembolism among factor V Leiden carriers: a community-based cohort study. J Thromb Haemost.

[8] Zöller B, Svensson PJ, He X, Dahlbäck B (1994). Identification of the same factor V gene mutation in 47 out of 50 thrombosis-prone families with inherited resistance to activated protein C. J Clin Invest.

[9] de Moerloose P, Wutschert R, Heinzmann M, Perneger T, Reber G, Bounameaux H (1998). Superficial vein thrombosis of lower limbs: influence of factor V Leiden, factor II G20210A and overweight. Thromb Haemost.

[10] Dentali F, Crowther M, Ageno W (2006). Thrombophilic abnormalities, oral contraceptives, and risk of cerebral vein thrombosis: a meta-analysis. Blood.

[11] Mahmoud AE, Elias E, Beauchamp N, Wilde JT (1997). Prevalence of the factor V Leiden mutation in hepatic and portal vein thrombosis. Gut.

[12] Zou Y, Zhang X, Zhang J, Ji X, Liu Y (2017). Factor V G1691A is associated with an increased risk of retinal vein occlusion: a meta-analysis. Oncotarget.

[13] Linnemann B, Schmidt H, Schindewolf M (2008). Etiology and VTE risk factor distribution in patients with inferior vena cava thrombosis. Thromb Res.

[14] Islam R, Kundu S, Jha SB (2022). Cirrhosis and coagulopathy: mechanisms of hemostasis changes in liver failure and their management. cureus.

[15] Islam H, Puttagunta SM, Islam R (2022). Risk of stroke with mitral stenosis: the underlying mechanism, treatment, and prevention. Cureus.

